# Osteosarcoma of the appendicular skeleton in dogs: consensus and guidelines

**DOI:** 10.3389/fvets.2025.1633593

**Published:** 2025-09-22

**Authors:** Gerry Polton, Juan F. Borrego, Francisco Clemente-Vicario, Craig A. Clifford, Dariusz Jagielski, Martin Kessler, Tetsuya Kobayashi, Didier Lanore, Felisbina L. Queiroga, Lucas Rodrigues, Annika Tranaeus Rowe, Péter Vajdovich, Philip J. Bergman

**Affiliations:** ^1^North Downs Specialist Referrals, Bletchingley, United Kingdom; ^2^Hospital Aúna Especialidades Veterinarias IVC-Evidensia, Valencia, Spain; ^3^La Merced Veterinary Specialists, Alicante, Spain; ^4^Bluepearl, Malvern, PA, United States; ^5^Faculty of Biological and Veterinary Sciences, Veterinary Institute, Nicolaus Copernicus University, Toruń, Poland; ^6^Department of Clinical Oncology, Tierklinik Hofheim, Hofheim, Germany; ^7^Japan Small Animal Cancer Center, Tokorozawa-City, Japan; ^8^Oncology Unit, Clinique Hopia, Guyancourt, France; ^9^CECAV, University of Trás-os-Montes and Alto Douro, Vila Real, Portugal; ^10^Estima Diagnósticos e Especialidades, Taubaté, Brazil; ^11^Evidensia Specialist Animal Hospital Strömsholm, Strömsholm, Sweden; ^12^Department of Physiology and Oncology, University of Veterinary Medicine, Budapest, Hungary; ^13^Veterinary & Cancer Immunotherapy Programs, Focused Ultrasound Foundation, Charlottesville, VA, United States

**Keywords:** osteosarcoma, guidelines & recommendations, canine, treatment, prognosis

## Abstract

Osteosarcoma (OSA) in dogs poses a clinical challenge to veterinary practitioners across the globe. As knowledge evolves, so too do clinical practices. However, there remain uncertainties and controversies. There is value for the veterinary community at large in the generation of a contemporary wide-ranging guideline document. The aim of this project was therefore to assimilate the available published knowledge into a single accessible referenced resource and to provide expert clinical guidance to support professional colleagues as they navigate current OSA challenges and controversies. Primary bone tumors are common in dogs. The history and clinical signs relate to the anatomic site of the tumor. Most canine patients present with a sudden-onset lameness that can appear to improve temporarily in response to analgesia and rest. Most patients do not have detectable metastasis at the time of diagnosis, but most canine patients do develop metastasis within months without appropriate therapy. Surgical resection using wide margins is currently the mainstay of therapy for the local control of the primary tumor. Most commonly, this comprises limb amputation in dogs, but not all dogs are considered good candidates. Anti-metastatic therapy is vital in dogs if surgery is going to offer a good chance of achieving a durable benefit. While there are many limb-sparing and palliative therapy options for dogs with OSA, most have not been shown to achieve superior outcomes compared with amputation and adjuvant chemotherapy. There is a role for radiotherapy in the palliative treatment of OSA. Immunotherapy should be considered a developing treatment modality—multiple immunotherapeutic approaches have yielded positive results in dogs in small experimental studies. It is hoped that this document will serve as a useful resource to practitioners all over the world, to help them better understand this disease and provide the best options for patients to extend quality of life and survival, either within the primary care or referral hospital setting.

## Introduction

OSA in dogs presents a dual challenge to the veterinarian, regardless of experience and facilities. Firstly, when arising in a weight-bearing bone, lesions are consistently painful. As a second challenge, primary osseous OSA is associated with a high prevalence of distant metastasis. Clinical stage provides the best indication of prognosis. There are many treatment strategies reported, which generally can be separated into treatments for the primary tumor and treatments for metastatic disease. The purpose of this guideline document is to provide a succinct yet comprehensive overview of OSA management in dogs.

## Methodology

Comprehensive literature reviews were carried out. Initial drafts of each section of the guidelines were written by individual authors. The guidelines were then combined into one document and reviewed by all authors. Areas of uncertainty or controversy were highlighted and associated recommendations and opinions were arrived at by group consensus. To quantify the strength of evidence available to support the information provided, references used to support statements were classified with a level of evidence (LOE) and overall evidence grade (OEG) as detailed in Elwood [(1) (LOE 2a OEG B)].

## Epidemiology and etiopathogenesis

OSA is the most common primary bone tumor in dogs, representing approximately 85% of all canine skeletal tumors [(2, 3) (LOE 3a–4a, OEG B)]. Risk factors for canine OSA include breed, size and age. A recent epidemiological study reported an overall 1-year OSA prevalence of 0.037%, with prevalences tenfold greater than baseline in the Scottish Deerhound (OR 118.4), Leonberger (OR 55.8), Great Dane (OR 34.2), Rottweiler (OR 26.7) and Greyhound (OR 11.9) [(4) (LOE 2c; OEG B)]. Increased risk is additionally reported in the Irish Wolfhound, Saint Bernard, Irish Setter, Old English Sheepdog, Bull Mastiff, Doberman Pinscher, German Pointer, Labrador Retriever, Lurcher, German Shepherd, Golden Retriever, Boxer and Landseer [(2, 4–11) (LOE 2c–4a, OEG B)]. In addition to larger breeds being affected, dogs weighing at or above the mean for their breed are 1.65 times as likely to develop OSA than those weighing below the breed mean [(4) (LOE 2c, OEG B)]. Similar associations are seen in humans, where increased birthweight, being taller than average as an adolescent or being a very tall adult increases the risk of OSA [(12) (LOE 2a, OEG B)]. While prevalence is greater among large- and giant-breed dogs, OSA has been shown to behave equally aggressively in smaller breeds [(13) (LOE 4b, OEG C)].

Appendicular limb OSA mainly affects middle-aged to older dogs, with studies reporting median ages of 7 to 9 years with a small first peak of incidence between 18 and 24 months [(2, 4, 8, 14–18) (LOE 2c–4b, OEG B)]. The early peak corresponds with the principal human peak of incidence in late puberty [(5, 12) (LOE 2a–5, OEG B)].

Older studies suggested that males are at slightly greater risk than females [(2, 8, 15, 19, 20) (LOE 4a, OEG C)]. However, this does not emerge as a risk factor once the greater weight of male dogs is accounted for [(4) (LOE 2c, OEG B)]. Similarly, causal associations between neutering status and OSA development are inconsistent [(3) (LOE 3a, OEG B)], with studies showing neutering increasing risk [(21, 22) (LOE 2c-3b, OEG B)] and not affecting risk [(4) (LOE 2c, OEG B)].

While OSA occurs most commonly in the axial skeleton in many species [(12) (LOE 5, OEG D)], appendicular OSA accounts for the majority of cases in dogs (69 to 86% of cases) [(2, 3, 5, 17, 23–25) (LOE 2c–4a, OEG B)] and humans (82 to 97% of cases) [(26–28) (LOE 2c–4a, OEG B)]. The vast majority of canine and human OSA arises within the medullary cavity, with tumors arising from the periosteum being rare in dogs [(29) (LOE 5, OEG D)] and humans [(30) (LOE 3a, OEG B)]. Both canine and human appendicular OSA occur predominantly within the metaphyseal region of long bones. The difference in the most commonly affected sites of bone tumors between humans and dogs is likely due to the distinct weight-bearing mechanics of the two species. In humans, appendicular OSA is most commonly found in the femur and tibia; in dogs where the forelimb supports 60% of the dog’s bodyweight, appendicular OSA is found twice as often in the forelimb (proximal humerus and distal radius) than the hindlimb (distal femur and proximal and distal tibia) [(2, 3, 6, 12, 27, 28) (LOE 2c–4a, OEG B)].

There is plentiful evidence implicating genetic and heritable factors for the development of OSA in dogs. Somatic mutations in tumor-suppressor genes associated with both human and canine OSA have been identified, including: TP53, MYC, PTEN, RUNX2, CDKN2A/B and DLG2 [(31–36) (LOE 3a–4b, OEG B)]. TP53 mutations have been reported to be present in around 60%, DLG2 mutations in 56% and SETD2 mutations in 21% of OSA tumor samples [(33, 34) (LOE 3b–4c, OEG B)]. Breed-associated inheritance of OSA has been described in multiple breeds [(3, 37, 38) (LOE 2b–3b, OEG B)]. OSA occurs much more frequently in dogs than in people, with a lifetime incidence risk 30 to 50 times higher in dogs [(5, 23) (LOE 5, OEG D)]. In larger dogs and humans, more cell divisions are needed for the formation and continuous remodeling of long bones, increasing the potential for mutations. Canine OSA tends to occur in major weight-bearing bones adjacent to late-closing physes where mitotic activity is greatest. Known drivers of osteoblast replication are associated with OSA development: fracture-associated OSA can develop secondary to the original trauma or implant placement, with a reported incidence of 0.08% [(39) (LOE 3b, OEG B)]; OSA is also a rare late complication of chronic osteomyelitis [(40) (LOE 5, OEG D)].

It has been proposed that the high rates of OSA in large- and giant-breed dogs may partly be explained by artificial selection for large breeds without co-selection for cancer-protective mechanisms that would occur under conditions of natural selection, as evidenced in whales and elephants, which both exhibit multiple differing species-specific tumor-suppressor genes [(12) (LOE 5, OEG D)].

## History and clinical signs

Dogs usually present with a history of intermittent weight-bearing lameness that worsens over time. A history of mild trauma before the onset of lameness is sometimes reported. Acute presentation for sudden, non-weight-bearing lameness is also possible with a pathological fracture [(3) (LOE 5, OEG D)]. This situation is estimated to occur in fewer than 3% of cases and can occur with or without initial trauma [(3, 41, 42) (LOE 3a–4b, OEG B)]. Pathological fracture is more common in certain breeds (Rottweilers, Irish Wolfhounds and Greyhounds), with lameness present in 60% of dogs preceding the fracture [(42) (LOE 4b, OEG C)]. Bone lysis is a risk factor [(41) (LOE 4a, OEG C)].

The main clinical signs induced by appendicular OSA are lameness, pain, and local swelling [(43) (LOE 5, OEG D)]. Metaphyseal swelling can be soft to firm and difficult to palpate in heavily muscled areas (e.g., the shoulder). Local pain is very variable. Initially, the clinical signs respond to treatment with nonsteroidal anti-inflammatory drugs (NSAIDs) or analgesics, and the dog may be misdiagnosed with an orthopedic or soft tissue injury. It is therefore essential to consider osteosarcoma (OSA) as a differential diagnosis in large- to giant-breed dogs presenting with lameness and pain localized to the metaphyseal region [(3) (LOE 5, OEG D)].

Patients with metastatic disease may have clinical signs referable to the site of metastasis—for example, pain associated with a bony metastasis. However, the clinical signs linked to pulmonary metastases are rarely respiratory, even with a large intrathoracic component. Rarely, pain and mild to moderate swelling of the distal limbs are associated with the unusual paraneoplastic syndrome of hypertrophic osteopathy associated with large intrathoracic metastases. More generally, the signs associated with significant metastasis are systemic, namely weight loss, decreased appetite, malaise, and lethargy [(3) (LOE 5, OEG D)].

### Epidemiology, etiopathogenesis, history and clinical signs: recommendations

- If an at risk breed patient is presented with acute-onset unilateral lameness, a detailed orthopaedic examination should be carried out, paying particular attention to OSA predilection sites by applying firm digital pressure to the long bone metaphyses.
- Diagnostic imaging is recommended at an early stage in patients with an at-risk signalment presenting with unexplained acute-onset or persistent or recurring lameness.
- Additional studies are required to understand the relationship between the timing of neutering and the risk of OSA development.

### Epidemiology, etiopathogenesis, history and clinical signs: opinion

- Unless or until strong evidence is presented to the contrary, we believe it is prudent to delay neutering in at-risk breeds until they have achieved skeletal maturity. This is an area of current controversy; knowledge and recommendations may change.

## Diagnosis

### Diagnostic imaging

**Figure 1:**
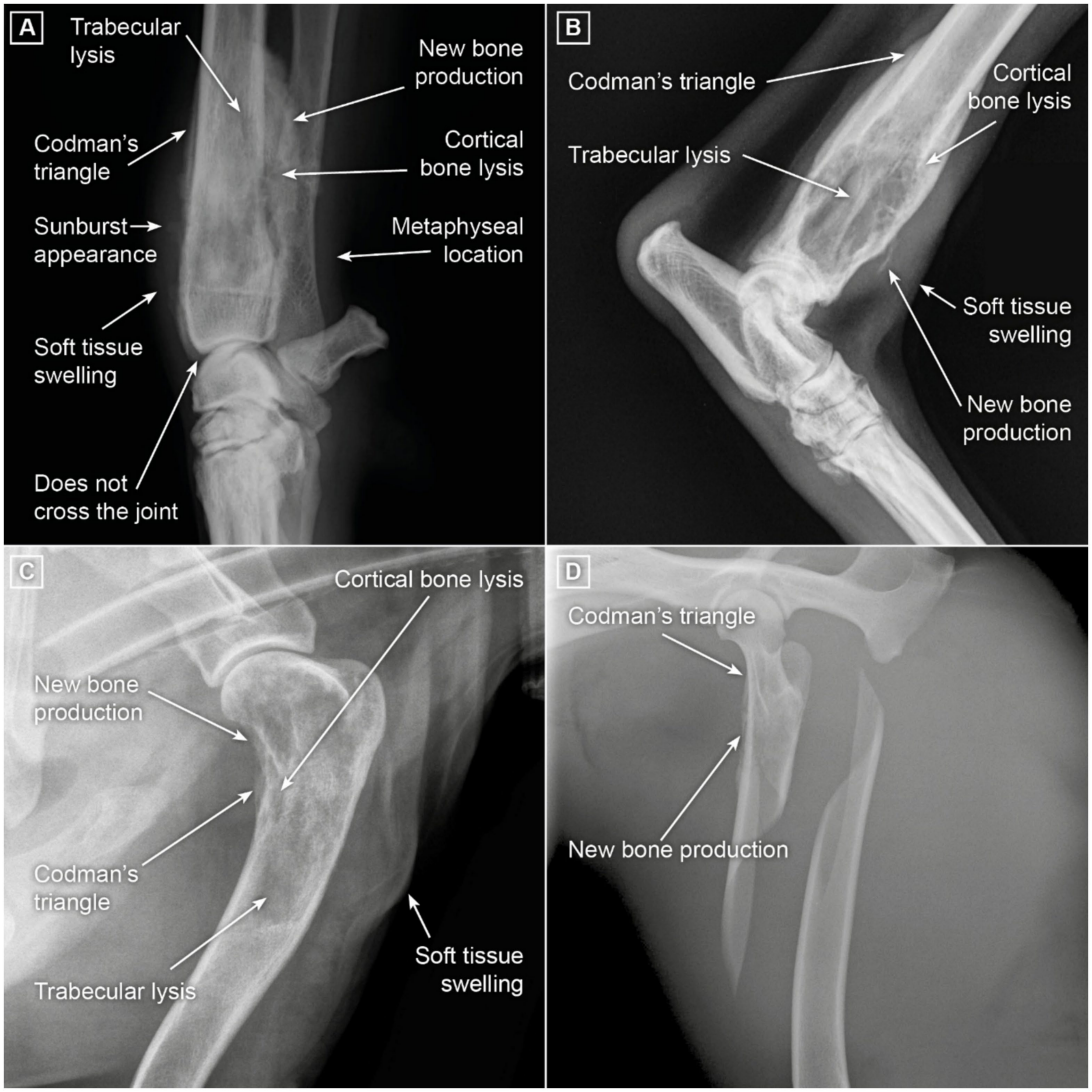
Radiographic images depicting typical signs of OSA. **(A)** Distal radial OSA. Osteolysis is apparent as thinning of the bold white cortex and loss or smudging of the fine internal trabecular pattern. Significant disordered new bone formation is evident proximally and caudally. On the cranial aspect, new bone formation is apparent where the periosteum has lifted (Codman’s triangle). When this new bone formation is rapid, bone fibers align perpendicular to the axis of the bone (Sharpey’s fibers) creating a sunburst appearance. Soft tissue thickening is recognizable in the distal limb. OSA typically arises in the metaphyseal region of the bone. It does not cross the joint (except occasionally when infiltrating the round ligament of the hip and extending into the acetabulum). **(B)** Distal tibial OSA. Changes are noted as in A. In this tumor, lytic changes are more apparent; there is less chaotic new bone formation. **(C)** Proximal humeral metaphysis is the second most common site of origin of OSA in dogs. **(D)** Proximal femoral metaphysis OSA is uncommon. Surrounding soft tissue can mask radiographic changes and reduce clinical impact, enabling the disease to progress further before clinical signs become apparent, for example, by pathological fracture.

Radiography remains a fundamental tool for detecting and staging canine OSA. The most common radiographic findings (see Figure 1) include cortical lysis (osteolytic), trabecular lysis (loss of trabecular pattern), bone production (osteoblastic), Codman’s triangle, and sunburst pattern into local soft tissues—generally at a metaphyseal location. It is usually accepted that OSA does not cross joints, with the exception of femoral head OSA, which may invade the acetabulum via the round ligament [(44) (LOE 4a, OEG C)].

Other causes of lytic bone lesions exist that can be challenging to differentiate from OSA using radiography alone (Table 1). Computed tomography (CT) is useful for questionable early cases, to demonstrate early medullary and/or cortical bone lysis, and for cases undergoing palliative irradiation to assess the degree of cortical destruction and thus risk of subsequent pathological fracture [(45) (LOE 4a, OEG C)]. However, CT does require sedation or general anesthesia [(46) (LOE 5, OEG D)] and is typically available only at larger primary care clinics or specialty centers.

**Table 1:** Differential diagnoses for canine OSA.

Condition	Key features	Radiographic appearance	Prognosis and treatment
Osteomas	Benign tumors, well-defined, radiodense; painless on palpation.	Radiographically dense and sharply defined.	Surgical excision is curative.
Bone cysts	Benign lesions, often in young animals; may resemble highly resorptive OSA.	Multilobular, sharply defined radiolucent defects.	Histological confirmation required; excision is curative.
Bacterial osteomyelitis	Associated with penetrating trauma (e.g., bites, open fractures).	Localized lysis and reactive changes.	Treat infection; surgical debridement if necessary.
Fungal osteomyelitis	Related to travel to endemic areas (e.g., *Coccidioides*, *Blastomyces*).	Monostotic, metaphyseal lytic lesions; lung infiltrates possible.	Antifungal therapy; histologic and serologic confirmation required.
Leishmania lesion	Often bilateral, osteolytic and osteoproductive lesions, lytic changes often span joints; consideration in endemic areas.	Periosteal and intramedullary proliferation; typically diaphyseal and related to the nutrient foramen.	Variable prognosis and management focusing on antiparasitic therapy, pain control, and supportive care, while monitoring for relapses.
Multiple cartilaginous exostosis	Developmental, hereditary, occurs in growing dogs; may undergo malignant transformation.	Benign trabecular radiographic pattern.	Surgical excision if clinical signs persist post-skeletal maturity.
Chondrosarcoma	Occurs in large- and medium-breed dogs; low metastatic potential, but up to 25% reported.	Proximal tibia common; less aggressive lytic or mixed changes.	Metastasis rates [(178) (LOE 4b, OEG C)]: Grade 1–10% Grade 2–31% Grade 3–50%
Fibrosarcoma	Rare, high metastatic rates in high-grade tumors; difficult to distinguish histologically.	Mixed lesions; similar to fibroblastic OS.	Complete resection can be curative in low-grade cases.
Primary hemangiosarcoma	Rare, highly metastatic; widespread metastasis within 6 months.	Predominantly lytic lesions.	Poor prognosis; 1-year survival rate: 28% [(179) (LOE 4a, OEGC)].
Solitary osseous plasmacytoma	Rare, benign, painful (75%), reported in tibia &carpal bones. More frequently axial.	Radiographically lytic lesions.	Radiotherapy reported, survival 545 and 798 days. Systemic progression possible. [(180) (LOE 4c, OEG C)].
Multiple myeloma	Uncommon. Malignant, painful. Axial always with concurrent appendicular involvement often. Paresis/paralysis often noted due to extradural compression.	Multiple large, lytic bony lesions. Clonal gammopathy. Plasmacytosis (>20%) in bone marrow. Light chain proteinuria.	Chemotherapy (melphalan, prednisolone); radiation therapy, pamidronate. Median survival time 384 days [(181) (LOE 4c, OEG C)].
Lymphoma	Rare. B-cell origin. Lameness, pain, pathologic fracture, anemia, thrombocytopenia and atypical circulating lymphocytes.	Osteolytic lesions in long bones, ribs and/or dorsal spinous processes. Typically bilateral. Can affect metaphyses.	Polyostotic: poor prognosis. Monostotic: radiation +/− systemic chemotherapy may achieve lasting remission [(182, 183) (LOE 4c, OEG C)].
Histiocytic sarcoma	Rare. Malignant. Solitary or disseminated. Axial or appendicular. Solitary typically periarticular. May have lymph node involvement. Bernese Mountain Dogs, Rottweilers, Golden retrievers predisposed.	Consistently osteolytic. Some also have productive components. Periarticular location highly suggestive.	Disseminated form, prognosis very poor. Localized tumor confers more favorable outcome using surgery, radiotherapy and/or chemotherapy (lomustine). [(184) (LOE 4c, OEG C)].
Metastatic bone cancer	Rare. Common origins: mammary, spleen and tonsil. Carcinoma (64%), hemangiosarcoma (20%). Multiple sites (38%). Mostly humeri and vertebrae.	Radiographically osteolytic, mixed and osteoproliferative/osteosclerotic lesions.	Prognosis very poor. Multiple systemic medical therapies reported without consistent success. [(185) (LOE 4b, OEG C)].

For patients undergoing limb-sparing techniques, advanced imaging modalities such as CT and magnetic resonance imaging (MRI) provide more detailed anatomical information, aiding in precise tumor localization and assessment of local tissue involvement [(47, 48) (LOE 4c, OEG C)]. Finally, for patients in which stereotactic body radiation therapy is to be considered, CT is imperative for both image-guided treatment planning and to determine its feasibility utilizing a new scoring scheme [(45) (LOE 4a, OEG C)].

### Clinical pathology

#### Cytology

Diagnosis of canine OSA requires microanatomic evaluation of tumor tissue, which is typically obtained via FNA and/or biopsy [(3, 49) (LOE 3a, OEG B)]. FNA, being less invasive, does not require general anesthesia and offers a less traumatic option for the dog [(49, 50) (LOE 3a–4b, OEG B)]. The reported sensitivity of cytology for diagnosing histologically confirmed OSA as sarcoma ranges from 70 to 97%; specificity ranges from 80 to 100% [(51–53) (LOE 4a–b, OEG C)]. However, while cytology can diagnose neoplasia, its effectiveness in definitively diagnosing OSA is imperfect [(52) (LOE 4b, OEG C)]. Cytochemical staining for ALP supports the differentiation of OSA from other bone tumors [(54–57) (LOE 4a–c, OEG C)], but this test is not widely accessible. Perhaps surprisingly, sensitivity of preoperative histopathology is lower than that of fine needle aspirate (FNA) cytology (45.5% versus 53.6% for histotype and 72.8% versus 100% for malignancy) [(53) (LOE 4a, OEG C)].

#### Blood work

In dogs with OSA treated with amputation and chemotherapy, higher numbers of circulating monocytes (>0.4 × 10^3^ cells/μL) and lymphocytes (>1.0 × 10^3^ cells/μL) were found to be associated with a shorter disease-free interval (DFI) [(58) (LOE 4a, OEG C)].

Although serum ALP cannot differentiate OSA from other bone-forming tumors or reactive bone lesions [(54) (LOE 4a, OEG C)], dogs with normal pre-treatment ALP levels (total ALP and ALP of bone origin) survived significantly longer than animals with increased pre-treatment ALP levels [(59–67) (LOE 2b–4b, OEG B)]. Serum ALP of bone origin correlates with osteoblastic activity [(68) (LOE 4a, OEG C)]. Human osteosarcoma tumour extracts have high activity of this enzyme [(69) (LOE 4b, OEG C)]. Elevated ALP is considered to correlate with micrometastatic disease or a greater tumor burden, both of which contribute to reduced survival [(67) (LOE 3b, OEG B)].

#### Inflammation

Dogs with OSA have an altered pro- and anti-inflammatory immunologic profile compared with healthy dogs regardless of NSAID use [(70) (LOE 3b, OEG B)].

#### Calcium homeostasis

Dogs with OSA showing high immunostaining intensity for PTHR1 had shorter average survival times (mean = 139 ± 27 days) compared with those with low immunostaining intensity (mean = 290 ± 68 days) [(71) (LOE 4b, OEG C)]. PTHR1 is a G-protein coupled receptor which acts as a regulator of cell growth and differentiation in developing tissues, for example skeletal growth plate [(72) (LOE 2a, OEG B)].

#### Biomarkers

Identification and quantification of plasma-derived, circulating tumor DNA by “liquid biopsy” is gaining traction as a non-invasive tool to aid diagnosis and guide treatment decision-making in human cancer patients; initial work indicates corresponding results in canine cancer patients [(73, 74) (LOE 2a–4c, OEG B)]. Several authors have evaluated various novel biomarkers belonging to cell signaling, bone development and other molecular pathways in an effort to better understand disease progression and support the development of targeted therapies [(31) (LOE 3a, OEG B)]. A detailed listing of these is outside of the scope of this article. Currently, the aforementioned ALP is the only widely available biomarker with clinical application.

### Biopsy and histology

Percutaneous core biopsy (often performed with a Jamshidi needle or bone trephine) obtains both cellular and matrix components. Therefore, as long as tumor tissue is harvested, it provides a more representative sample of the tumor than FNA, increasing the likelihood of accurate histopathological characterization. Typically conducted under heavy sedation or, preferably, general anesthesia, it relies on imaging techniques for accurate tumor localization [(75–77) (LOE 3a–4b, OEG B)]. Alternative biopsy methods include incisional and excisional biopsies [(78) (LOE 5, OEG D)]. While incisional biopsies provide ample tissue for diagnosis, they carry a modest risk of fracture, and also confer risk of bleeding, infection, and potential for tumor spread [(3) (LOE 3a, OEG B)]. Excisional biopsy involves complete tumor removal, facilitating an extensive evaluation but is limited by the need for complete resection and carries its own set of risks, including local recurrence when limb-preserving techniques are applied [(79) (LOE 4a, OEG C)].

Biopsy site selection is vital to avoid false negatives. A recent study reported 27.8% (5/18) of malignant lesions were not identified histologically, with 4 of the 5 diagnostic errors due to a diagnosis of reactive bone tissue instead of a neoplastic process [(51) (LOE 4a, OEG C)]. For bone tumors, targeting the lesion’s center is recommended to avoid the less diagnostic periosteal reaction found at the periphery [(3) (LOE 3a, OEG B)]. Advances in imaging, such as CT and magnetic resonance imaging (MRI) guided biopsies, have enhanced the precision of biopsy procedures, minimizing sampling errors and ensuring the evaluation of tumor sections most representative of the disease [(75, 76) (LOE 3a–4b, OEG B)].

**Figure 2:**
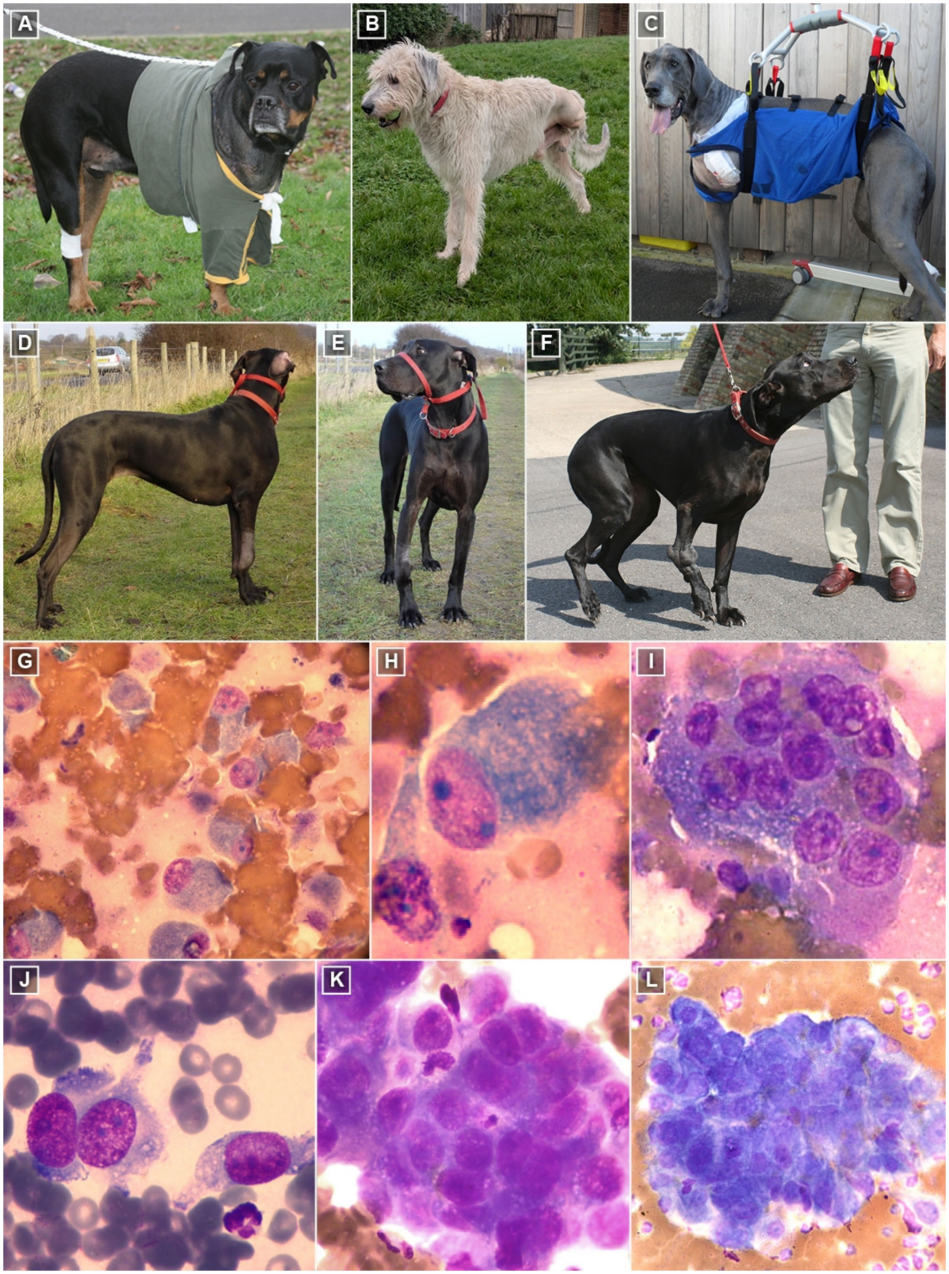
Clinical and cytological features of canine OSA. **(A–C)** Dogs are generally ambulatory within 24 h of amputation. Aids to ambulation are available and may be particularly advantageous in the immediate post-operative period. **(D,E)** Dog with distal radial OSA weight-bearing but favoring affected limb 4 weeks after hypofractionated radiotherapy. **(F)** Same dog 2 years later, non-weight-bearing lame with chronic pathological fracture. **(G,H)** German Shepherd, 11 years old. **(G)** OSA of chondroblastic or osteoblastic type. Large blast cells showing moderate anisocytosis are visible with granular basophilic cytoplasm and excentric nuclei. The cytoplasm contains light metachromatic granules. These might be chondroid material. The nuclei are round to oval in shape, with large and prominent basophilic nucleoli and a clumped chromatin pattern. Diff-Quik staining (400×). **(H)** Chondroblastic or osteoblastic cell with multiple prominent nucleoli, and a cell (left) showing mesenchymal patterns. Quick-diff staining (1,000×). **(I)** Multinucleated cell that contains multiple uniform nuclei with prominent single nucleoli. The cytoplasm is basophilic and often contains eosinophilic or lighter basophilic granules; this is consistent with a large osteoclast. The background is pinkish, which seems to be osteoid matrix. Diff-Quik staining (1,000×). **(J)** Bone lesion in an 8-year-old crossbreed. OSA of fibroblastic type. Large spindle-shaped cells with cytoplasmic projections at the poles of the cells. The cytoplasm is granulated and contains small vacuoles. Nuclei are oval in shape with a coarse chromatin pattern and moderately large, prominent nucleoli. Diff-Quik staining (1,000×). **(K,L)** 7-year-old Doberman Pinscher. **(K)** Bone lesion. Large osteoblasts or chondroblasts showing marked anisocytosis and anisokaryosis are visible, with granular basophilic cytoplasm and eccentric nuclei. Nuclear molding is also visible. The cytoplasm contains light metachromatic granules. These might be chondroid material. The nuclei are round to oval in shape, with large, prominent, dark basophilic nucleoli and a highly clumped chromatin pattern. Diff-Quik staining (1,000×). **(L)** Pulmonary lesion. The cells are cohesive like the primary bone lesion, although anisokaryosis and cytoplasmic basophilia are more marked. Note that there are several granulocytes in close proximity to the tumor mass. Diff-Quik staining (1,000×).

Canine appendicular OSA can present with diverse cell morphologies and patterns and, depending on its anatomic localization, be classified as central (arising from the medullary cavity) or surface (arising from the periosteum) [(29, 49) (LOE 3a, OEG B)]. The central form can be further classified into 6 histological subtypes (osteoblastic, chondroblastic, fibroblastic, telangiectatic, giant cell, and poorly differentiated) with mixed subtypes commonly observed within the same tumor. Histologically, OSA can sometimes be confused with a callus repair, and one must look for maturation within the callus as a differentiating feature in the cell population [(49, 80) (LOE 5, OEG D)]. This variability underscores the complexity of OSA and the importance of thorough histopathological examination for accurate diagnosis and treatment planning. Clinical and histological features of OSA can be seen in Figure 2.

### Diagnosis: recommendations

- Consider use of CT for suspected OSA occurring in atypical anatomical sites with unusual radiographic or clinical findings, and in areas endemic for infectious causes of bone lesions (e.g., Leishmania).
- Cytology is recommended as the first tissue sampling method of choice. Note that it is not always diagnostic, as OSA is often not readily accessible.
- Avoid biopsy if planning radiation as this further undermines bone integrity. Experienced oncologists will rely on the combination of clinical, radiological and cytological findings, and knowledge of endemic pathogens which might radiographically mimic OSA.
- Avoid biopsy in cases with a “typical” presentation as it predisposes to a pathologic fracture and “outgrowth” of the tumor through the biopsy canal.

### Diagnosis: opinions

- Provided the owner is adequately informed of the associated risks, it is acceptable to establish a diagnosis via histopathology following amputation, when signalment, clinical presentation and radiologic findings are compatible with osteosarcoma.
- Many oncologists only seek to make a preoperative diagnosis when lesions are subtle or inconsistent with typical primary bone tumors, and then preferring to rely on cytology in the majority of cases.
- Biopsy typically requires general anesthesia, whereas FNA can often be performed under sedation; in some cases local analgesia may suffice. Ultrasonography can aid in identifying cortical bone defects, which may serve as optimal target sites for aspiration.
- At this point in time there aren’t enough validated data to support the use of liquid biopsy results in clinical decision making in dogs with OSA. As the quality and quantity of baseline data from circulating tumour DNA analysis increase, liquid biopsy might gain a role in clinical management of canine primary bone tumours.

## Clinical staging

Tumor staging is recommended and should include orthopedic examination and local lymph node cytology for bone and lymph node metastasis, respectively [(3) (LOE 3a, OEG B)]. Less than 5% of dogs have lymph node metastasis, but it is a negative prognostic factor [(81) (LOE 4a, OEG C)]. Three-view chest radiographs are recommended to ensure no overt evidence of pulmonary metastasis, which is noted in approximately 5% of cases [(3, 46, 82, 83) (LOE 3a–4b, OEG B)]. The use of thoracic CT offers a more sensitive assessment of the lungs for staging purposes [(46, 82) (LOE 4b, OEG C)]. Additionally, nuclear medicine techniques such as bone scintigraphy and positron emission tomography (PET) can contribute to the detection of metastatic lesions, providing critical information for treatment planning and prognosis. Currently, availability of these techniques is mostly limited to academic centers [(83–86) (LOE 4a–5, OEG C)].

There is a surgical staging system for humans with sarcoma of the skeleton. The system is based on histological grade (G), anatomic setting of primary tumor (T) and metastasis (M). Three stages are described:

Stage 1: no signs of metastasis (M0), low histologic grade (G1).
Stage 2: no signs of metastasis (M0), high histologic grade (G2).
Stage 3: signs of regional or distant metastasis (M1), regardless of histologic grade.

Stages 1 and 2 are subdivided by anatomic setting into group A where the tumor remains within the bone [intracompartmental (T1)] and group B where the tumor extends beyond the bone into other structures [extracompartmental (T2)] [(3, 87) (LOE 3a–4a, OEG B)].

Most canine cases would be stage 2B at diagnosis [(3) (LOE 3a, OEG B)]. However, this grossly underestimates the prevalence of metastatic disease, with approximately 90% of dogs having micrometastatic disease at the time of diagnosis [(82) (LOE 3a, OEG B)].

### Clinical staging: recommendations

- Detailed thoracic imaging is imperative to understanding the clinical stage of disease.
- Detailed examination is important to highlight atypical sites of potential cancer spread—for example, second skeletal sites and locoregional lymph nodes.
- CT, especially with contrast media, provides greater accuracy than radiography in determining the anatomic extent of disease.
- Examination of imaging studies by an experienced radiologist will improve discovery of clinically relevant findings that might otherwise be missed.

### Clinical staging: opinions

- While it might be intuitive that treatment decisions made with CT-derived clinical stage data would be superior to those made without, there is currently a lack of evidence to support this in veterinary patients.
- Advanced imaging (CT/MRI/scintigraphy) is not a prerequisite for rational definitive therapy.

## Osteosarcoma treatment in dogs

The principal 2 goals of treatment for OSA in dogs are relief of pain directly associated with the primary tumor and the control of metastatic disease. Pain management is always prioritized.

### Surgical treatment

#### Amputation

This is now widely regarded as the therapy of choice for appendicular OSA, with the exception of early-stage OSA arising from the distal metaphysis of the radius (see below) or the diaphysis of the ulna [(88) (LOE 4b, OEG C)] where limb-preserving surgical options may be appropriate. Amputation should achieve complete resection of the tumor and alleviation of the tumor-associated bone pain. In the case of tumors of the forelimb, amputation including the scapula or through the shoulder joint is recommended. In the hind limb with tumors distal to the stifle, amputation in the proximal femoral third or by disarticulation in the hip joint can be performed. In tumors arising from the distal femur, the level of amputation should be the hip joint. If the tumor extends into the femoral head, further extension into the acetabulum via the round ligament of the hip is possible, and partial pelvectomy (acetabulectomy) must be considered [(3, 89, 90) (LOE 3a–4a, OEG B)].

Contraindications for amputation are gait-compromising neurological disorders or relevant orthopedic deficits in the remaining limbs. Amputation is rejected by many pet owners and some veterinarians, although studies have shown good mobility even in large- and giant-breed dogs [(3, 91) (LOE 3a–4a, OEG B)]. Dogs that are non-weight-bearing lame have already demonstrated their ability to ambulate on three legs. Preoperative counselling of pet owners, supplemented with educational resources such as videos of three-legged dogs or testimonials from their caregivers, may help set realistic expectations and facilitate informed decision-making regarding the procedure. Despite the elimination of the primary tumor by amputation, this surgery remains palliative due to the high prevalence of undetected micrometastases. Amputation alone (or limb-sparing techniques) without subsequent chemotherapy results in survival times of only 3 to 4 months [(15, 19) (LOE 4a, OEG C)].

Today, orthopedic companies offer bespoke prostheses for distal limbs. In cases of tumors located distal to the hock or the carpus, such prostheses offer an alternative to total limb amputation. Prosthetic devices that induce bone tissue integration have been described [(92) (LOE 4c, OEG C)], but their use remains experimental.

#### Distal radial osteosarcoma limb-sparing

If the dog is a poor candidate for amputation due to underlying neurological or orthopedic conditions, or if it is declined by the owner for ethical or personal reasons, limb-sparing surgery may be considered as an alternative. Many different techniques have been described. Allogeneic bone grafts [(93, 94) (LOE 2b–4c, OEG B)], reimplantation of the patient’s own bone after extracorporeal destruction of the tumor [(95–98) (LOE 4c, OEG C)], and metallic endoprostheses [(94) (LOE 2b, OEG B)] can serve as a non-living substitute for the tumor-bearing part of the bone [(93, 96) (LOE 4c, OEG C)]. However, regardless of the technique, limb-preserving surgery does not produce better survival outcomes than amputation. After limb-preserving bone graft surgery in 220 dogs, local recurrence occurred in 25% of cases and infection in 44% of cases over a period of 1 year [(99) (LOE 4a, OEG C)]. In another study, 78% of the patients developed an infection, 36% an implant complication, and 24% a local recurrence. Metastases formed in 67% of patients. The median survival was 289 days [(100) (LOE 4b, OEG C)]. Complications might lead to the need to amputate, and a study evaluating dogs with failed limb-sparing surgery described a median survival time of 205 days following amputation [(101) (LOE 4a, OEG C)].

Stereotactic irradiation is a limb-sparing alternative to segmental tumor resections and grafting techniques [see Radiotherapy section].

#### Surgical treatment of metastases

Surgical removal of lung metastases has been reported in humans and dogs [(102–104) (LOE 4a–b, OEG C)]. Prerequisites for resection of lung metastases are a good general condition of the patient, slow progression of metastases (preferably a remission period >300 days) and the absence of evidence of tumor at other sites. Resection is only beneficial if the mediastinum and chest wall are not affected and the number of lung metastases does not exceed 2 radiographically visible nodules [(3, 103, 104) (LOE 3a–4b, OEG B)]. The published work reported cases imaged using x-ray. The impact of radiographically-undetectable but CT-detectable pulmonary nodules is unknown.

Applying these criteria, MST was 255 days after metastasectomy, compared with 49 days after the detection of lung metastases in cases without metastasectomy [(103) (LOE 4a, OEG C)]. In an older study with 36 dogs, the mean survival time after lung metastasis resection was 176 days [(104) (LOE 4b, OEG C)]. Usually, metastasectomy is performed by lateral or ventral thoracotomy but, in selected cases, thoracoscopic removal may be employed.

### Radiotherapy

Radiotherapy (RT) can be used for palliation of pain or for long term control of the primary tumour. The killing of tumor cells or the inhibition of osteoclast-mediated osteolysis are factors that may contribute to pain reduction in either scenario. Stereotactic body radiation therapy (SBRT) involving the administration of high-dose fractions of radiation (20 to 30 Gy) to the target site has emerged as a forefront technique, offering a balance between effective tumor management and minimally invasive treatment [(105) (LOE 3a, OEG B)]. This method is particularly significant for limb-sparing in cases where amputation or more aggressive surgeries are not viable options.

Pathologic fractures post-SBRT have been the main complication when using this type of treatment [(45, 106) (LOE 4a, OEG C)]. A recent study involving 123 dogs treated with SBRT at 130 anatomic locations revealed that the majority of dogs showed improvement in lameness within 3 weeks post-treatment [(107) (LOE 4a, OEG C)]. However, pathologic fractures developed in 41% of the cases. Just over half of these cases subsequently required limb amputation. The MST for dogs receiving SBRT was 233 days. It is interesting to note that a longer MST was observed in dogs that subsequently underwent amputation (346 versus 202 days).

Pathological fractures commonly occur in specific anatomical regions such as the distal tibia and proximal femur. A study looking at a CT-based scoring system assessed the risk of fractures in dogs undergoing SBRT, trying to evaluate different factors such as the degree of bone lysis and the length of the affected bone, providing a predictive framework for fracture risk [(45) (LOE 4a, OEG C)]. More recently, the measurement of the patient’s total body volume was significantly associated with pathological fractures following SBRT [(108) (LOE 4b, OEGC)].

Palliative RT consists of the use of hypofractionated protocols with the aim to relieve cancer induced pain. Multiple protocols have been described, with 4 weekly administrations of fractions of 6–8 Gy being a frequently used prescription. These protocols are typically well tolerated with minimal early toxicities, although RT can potentiate the risk of pathologic fractures in the long term. A recent study compared fracture rates in dogs receiving highly fractionated versus coarsely fractionated RT [(109) (LOE 4a, OEG C)]. Theoretically, hypofractionated protocols should have a higher associated risk of late complications, including bone necrosis, which predisposes to pathologic fracture. In contrast, the study reported that in fact the risk was higher when using more finely fractionated protocols. The authors concluded this might be due to poorer tumour control when using a lower dose per fraction, as tumour progression probably plays a major role in this complication as well. In this study, none of the dogs treated with hypofractionated RT combined with zolendronate developed fractures, and the authors suggested this drug could play a role in preventing fractures in dogs treated with RT. Multiple studies have evaluated the impact of bisphosphonates in outcome when treating dogs with palliative RT, with conflicting results, and further research is required to determine their role in this setting [(109) (LOE 4c, OEG D); (110) (LOE 4a, OEG C); (111) (LOE 4c, OEG D)]. Carboplatin has been proposed as a radiation sensitizer [(112) (LOE 5, OEG D)], although one study did not find its concurrent use in dogs receiving palliative RT had a statistical effect in survival. Additionally, severe toxicities have been described in dogs receiving concomitant carboplatin and RT, with grade 3 or 4 neutropenia and thrombocytopenia and grade 3 or 5 gastrointestinal toxicosis in 20 and 10% of dogs, respectively [(113) (LOE 4a, OEG C)]. Therefore, carboplatin is not routinely used as a radiation sensitiser for canine osteosarcoma.

Research into specialized limb-sparing techniques for specific tumor locations, such as ulnar ostectomy combined with SBRT, has shed light on limb preservation [(114) (LOE 4b, OEG C)]. Meanwhile, Nolan et al. [(115) (LOE 4a, OEG C)] investigated the impact of radiation dose and baseline pain levels on survival in dogs undergoing RT with or without chemotherapy. Higher radiation dose (SBRT vs. conventional hypofractionated RT) and lower pain score at diagnosis were associated with improved survival outcome. Their findings underline the importance of personalized treatment plans, considering individual pain management and disease characteristics.

Despite these advancements, the management of OSA using RT is not without challenges. The risk of pathologic fractures following SBRT remains a significant concern, necessitating careful patient selection and thorough diagnostic assessment. The potential for late side effects in tissues with slow turnover, such as bone, also warrants cautious consideration. These side effects can include tissue fibrosis, necrosis and loss of function, although they are less likely in acute pain palliation settings.

The treatment of canine appendicular OSA through RT, particularly SBRT, is evolving, with ongoing research enhancing our understanding and capabilities. The integration of advanced diagnostic tools, combined treatment modalities, and personalized patient care continues to shape the future of this technique, striving to improve the quality of life and survival outcomes for these patients.

### Medical therapy

#### Adjuvant medical treatment

Definitive-intent treatment of canine patients with OSA consists of achieving local control using various surgical or radiological techniques and, for patients at high risk of metastases, systemic chemotherapy. Adjuvant chemotherapy, when following surgery and/or RT, is considered the standard of care. Local treatment without chemotherapy typically provides patients with appendicular OSA with an MST of approximately 4 to 5.8 months and almost 90% mortality rate within 1 year, because of occult micrometastases at the time of diagnosis [(15, 19, 116, 117) (LOE4a–b, OEG C)]. Depending on the study and specific protocol, chemotherapy has improved MST to between 8 and 14 months. Most studies have evaluated carboplatin, doxorubicin and cisplatin used alone or in combination, usually in 4 to 6 doses, but no single protocol has been proven to be of greater benefit [(63, 115–125) (LOE 2b–4b, OEG B)]. Selected chemotherapy protocols are listed in Table 2.

**Table 2:** Treatment protocols for dogs with OSA.

Dose interval (weeks)	Starting protocol	No. of cycles	No. of dogs	Progression free survival (days)	Overall survival (days)	1-year survival	2-year survival	References
Carboplatin 300 mg/m^2^								
3	7 days before surgery	3–4	41	123	215	–	–	[(119) (LOE 4a, OEG C)]
	≤7 days after surgery	4	48	257	321	35.4%	–	[(118) (LOE 2b, OEG B)]
	After surgery	3–4	114	272	388	–	–	[(119) (LOE 4a, OEG C)]
	≤14 days after surgery	4	109	282	299–304	35%	19%	[(120, 126) (LOE 2b–4a, OEG B)]
	After surgery, majority ≤14 days	6	181	399–425	306–479	38%	13%	[(120, 122, 126) (LOE 2b–4a, OEG B)]
Doxorubicin 30 mg/m^2^								
2	27 or 41 days before surgery	5	35	–	366	50.5%	9.7%	[(186) (LOE 4b, OEG C)]
2	14 days after surgery	5	124	269	252	42%	15%	[(120) (LOE 2b, OEG B)]
2	14 days after surgery	5	303	–	240	35%	17%	[(63) (LOE 2b, OEG B)]
3	14 days after surgery	5	65	302	241	29%	14%	[(120) (LOE 2b, OEG B)]
Concurrent cisplatin 60 mg/m^2^ and doxorubicin 15–25 mg/m^2^								
2	2 days after surgery	–	53	–	345	–	–	[(124) (LOE 2b, OEG B)]
	10 days after surgery	–	49	–	330	–	–	[(124) (LOE 2b, OEG B)]
Alternating carboplatin and doxorubicin								
3	10–14 days after surgery	3 each	32	227	320	48%	18%	[(121) (LOE 4b, OEG C)]
	After surgery, majority ≤14 days	3 each	166	135–302	258–314	44%	20%	[(120, 122, 127) (LOE 2b–4b, OEG B)]
Sequential doxorubicin (3 doses q2) and carboplatin (3 doses q3)								
2	After surgery	6	38	–	317	43.2%	13.9%	[(128) (LOE 4b, OEG C)]
Comparison of interval between amputation and adjuvant therapy for a mix of treatment protocols: carboplatin 300 mg/m^2^ (*n* = 107) or doxorubicin 30 mg/m^2^ (*n* = 12) or cisplatin 70 mg/m^2^ (*n* = 11) or alternating (cisplatin *n* = 25 or carboplatin *n* = 8) and doxorubicin or cisplatin and dacarbazine 200 mg/m^2^/day for 5 consecutive days (*n* = 4) or alternating carboplatin and epirubicin 30 mg/m^2^ (*n* = 1)								
–	<5 days after surgery	–	52	375	445	–	–	[(125) (LOE 4a, OEG C)]
–	>5 days after surgery	–	116	202	239	–	–	[(125) (LOE 4a, OEG C)]

For platinum agents, carboplatin is considered the drug of choice. This is due to its lower risk of adverse events such as nephrotoxicity, nausea, and gastrointestinal toxicity compared with cisplatin which requires aggressive saline diuresis to minimise the risk and magnitude of kidney damage. When used as an adjuvant or neoadjuvant combined with amputation, carboplatin given every 3 weeks resulted in a median DFI of between 137 and 257 days and a MST of between 277 and 321 days [(118, 119, 126) (LOE 2b–4a, OEG B)]. Aggressive saline diuresis is necessary to minimize the risk of nephrotoxicity associated with cisplatin administration. Doxorubicin has been described as a monotherapy for adjuvant therapy for OSA. A 2-week interdose interval was required to demonstrate efficacy [(63, 124) (LOE 2b, OEG B)]. Treatment can be associated with myocardial toxicity; therefore, some oncologists recommend an echocardiographic examination before starting this drug, particularly in high-risk breeds [(63) (LOE 2b, OEG B)]. A lower proportion of dogs with chemotherapy-related adverse effects was observed when treated with single-agent carboplatin compared with single-agent doxorubicin [(120) (LOE 2b, OEG B)].

Dual-agent chemotherapy protocols with alternating or concurrent use of drugs do not appear to offer any survival advantage compared with single-agent protocols [(121, 122, 127–131) (LOE 2b–4b, OEG B)]. Chemotherapy is usually started at the time of suture removal but could be initiated before (i.e., neoadjuvant) or up to 3 weeks after limb or tumor removal [(119, 124) (LOE 2b–4a, OEG B)]. Neoadjuvant chemotherapy is standard of care in human medicine, with markedly improved rates of disease progression due to local recurrence and distant metastasis compared to controls. Improved outcomes are attributed to massive necrosis of the primary tumour and successful earlier targeting of micrometastases [(132) (LOE 1a, OEG A)]. In dogs, neoadjuvant chemotherapy resulted in statistically-insignificantly poorer survival [(119) (LOE 4a, OEG C)]. However, some data suggest early initiation of adjuvant chemotherapy up to 5 days after amputation of the affected limb does confer a survival advantage [(118, 125) (LOE 2b, OEG B)].

Metronomic chemotherapy (cyclophosphamide, meloxicam or piroxicam) has failed to yield a survival advantage when used either concurrently with adjuvant maximum tolerated chemotherapy, or as a maintenance therapy following amputation and conventional chemotherapy [(133–135) (LOE 2b–4b, OEG B)]. Toceranib phosphate as an adjuvant agent in dogs with appendicular OSA after amputation and 4 doses of carboplatin was added to metronomic chemotherapy (cyclophosphamide and piroxicam) [(133) (LOE 2b, OEG B)] or used as a single drug [(136) (LOE 2b, OEG B)], with no evidence of a survival benefit. Metronomic administration of lomustine following palliative RT has also been investigated in dogs with appendicular OSA and found to confer no benefit [(137) (LOE 4a, OEG C)].

#### Medical treatment of gross metastatic disease

There is currently no standard of care for dogs with advanced-stage OSA. Partial remission in 25% and stable disease lasting 8 weeks or more in 25% of dogs with measurable pulmonary metastases have been described in response to the combination of losartan and toceranib [(138) (LOE 2b, OEG B)] (see Immunotherapy section). Otherwise, treatment of gross measurable disease with conventional cytotoxic drugs or tyrosine kinase inhibitors is unrewarding. The MSTs of dogs with distant metastases range between 55 and 94 days when all sites and medical-only treatments are considered together [(139–142) (LOE 2b–4b, OEG B)]. It has been noted that dogs with bone metastases treated with RT and chemotherapy (130 days) [(139) (LOE 4a, OEG C)] and dogs with cutaneous/subcutaneous metastases treated with chemotherapy and surgical excision (94 days) appeared to have longer survival than others in those particular cohorts [(142) (LOE 4b, OEG C)]. Initial studies of toceranib phosphate efficacy in dogs with metastatic OSA delivered encouraging results [(143) (LOE 4a, OEG C)]; however, subsequent studies showed clinical benefit in only 10 and 17.6% of patients, with a median PFS of 36 and 57 days, respectively [(140, 141) (LOE 2b–4b, OEG B)]. Similarly, zoledronate used for patients with pulmonary OSA metastases had limited effectiveness (median PFS 28 days, median stage III-specific survival 92 days) [(144) (LOE 2b, OEG B)].

#### Medical palliative treatment

Dogs not considered suitable for definitive treatment can be offered palliative therapy. A multimodal approach is best, which may include analgesics, tumor ablation with radiation and chemotherapy, and inhibition of osteolysis. Combining chemotherapy with palliative RT in dogs with OSA may improve bone pain control and/or survival time [(110, 111, 145, 146) (LOE 4a–b, OEG C)].

**Figure 3:**
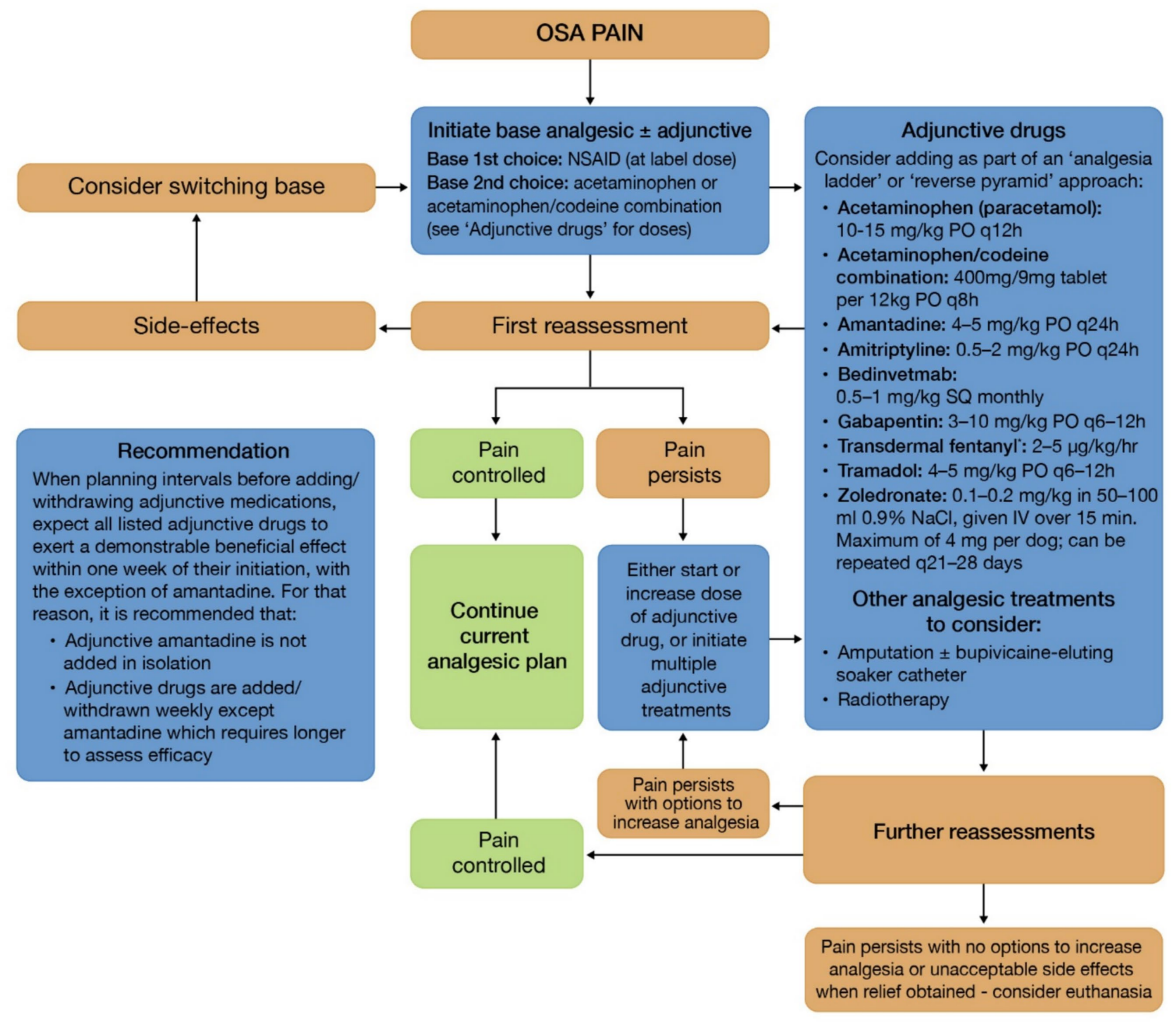
OSA analgesia decision tree. Adapted from WSAVA Global Pain Management Protocol: Cancer-related pain, 2022 WSAVA guidelines for the recognition, assessment and treatment of pain and table 16.4 in Supportive Care for the Cancer Patient [(175–177) (LOE 2c, OEG B)]. OSA dogs have a generalized increased pain sensitivity throughout their body with inflammatory and neuropathic components; therefore, a multimodal drug approach using a base analgesic and adjunctive analgesics as required to control OSA pain is recommended. Two approaches to OSA pain management include the “analgesia ladder’,” whereby there is a gradual addition of analgesic medications until adequate comfort is achieved, and the “reverse pyramid,” whereby there is an initial aggressive multimodal approach to “wind down” pain followed by sequential withdrawal of analgesic medications, providing comfort is maintained. Many of the adjunctive analgesic drugs used to control OSA pain are used off-label; prescriptions should be based on an individual risk–benefit assessment. *Long-term use is limited by the need to change fentanyl patches every 4 to 7 days. Clinicians should be aware of the abuse potential and danger to children when considering prescribing fentanyl patches.

A discussion of the importance of specific classes of analgesics in patients with OSA is beyond the scope of this paper, but readers are directed to Figure 3, which is adapted from widely used and referenced sources. The use of NSAIDs, opioids, local anaesthetics, NMDA antagonists, anticonvulsants, tricyclic antidepressants, aminobisphosphonates, corticosteroids and anti-NGF monoclonal antibodies should be considered. The drug of first choice for long-term management of bone cancer pain should be a COX-2 inhibitor, given reports of COX-2 overexpression in canine OSA. Furthermore, the MST of definitively treated patients undergoing limb amputation and chemotherapy can likely be prolonged by intensive perioperative analgesic treatment with both NSAIDs and a local anesthesia-eluting soaker catheter placed at the amputation site [(123) (LOE 4a, OEG C)]. Newer pain medications such as the anti-NGF monoclonal antibody tanezumab have also been shown to be effective in reducing bone cancer pain in humans [(147) (LOE 2b, OEG B)]. There is a canine anti-NGF agent, bedinvetmab, which is licensed for the treatment of pain associated with osteoarthritis.

Aminobisphosphonates are osteoclast inhibitors used in both human and veterinary medicine to treat bone cancer pain via inhibition of bone resorption and mechanical stabilization. Multiple studies have indicated their analgesic effect in dogs with appendicular OSA [(148–150) (LOE 2b–4c, OEG B)] although further studies are required to evaluate the impact of administration of bisphosphonates on outcome for dogs with OSA also receiving RT as conflicting results have been reported so far [(109–111, 151) (LOE 2b-4c, OEG B-C)].

#### Immunotherapy

Different immunotherapy options have historically been used to treat canine OSA. The best-documented used liposome-encapsulated muramyl tripeptide-phosphatidylethanolamine (L-MTP-PE) and was proven to be clinically efficacious for dogs with OSA [(152) (LOE 2b, OEG B)]. Despite their limitations and challenges, next-generation immunotherapies are showing promising results. These include cancer vaccines, checkpoint inhibitors, targeted therapies, adoptive cell therapies, and advanced molecular approaches, as well as their combinations.

Although previously reported in the literature, OSA cancer vaccines are currently gaining more attention. Vaccines aim to stimulate the immune system to target and destroy cancer cells. The use of *Listeria*-based vaccines [(153) (LOE 2b, OEG B)] and ErbB (EGFR/HER2)-specific vaccines [(154) (LOE 2b, OEG B)] have led to extended survival in initial canine studies. The ErbB-specific vaccine demonstrated *in vitro* inhibition of tumor growth, as well as inducing complete radiographic resolution of pulmonary metastases in 3 out of 3 immunized cases. However, the *Listeria*-based commercially approved vaccine failed to yield improvements in disease-free interval or overall survival (although immune response was able to differentiate elite from short-term survivors) [(155) (LOE 4a, OEG C)] and has since been removed from the market because some patients developed vaccine-specific *Listeria* infections, which represented a possible zoonotic threat [(156) (LOE 4b, OEG C)]. Another approach involves peptide-based vaccines, with some showing effectiveness in non-metastatic appendicular OSA compared with historical controls [(157, 158) (LOE 2b, OEG B)].

The recently reported clinical benefit induced by losartan when combined with toceranib for treatment in dogs with stage III osteosarcoma is thought to be mediated by blockage of monocyte recruitment and subsequent angiogenesis in dogs with metastatic OSA [(138) (LOE 2b, OEGB)]. The chimeric human/dog-DNA vaccine targeting CSPG4 has also shown potential in OSA treatment [(159) (LOE 2b, OEG B)]. Another approach used to break tumor tolerance is a combination of immunocytokines with immunomodulating radiation to induce antitumor immunity. This method is notable for its synergy between *in situ* vaccination and targeted radionuclide immuno-RT [(160) (LOE 4c, OEG C)].

Checkpoint inhibitors are pivotal in advancing canine cancer treatment. PD-1 inhibitors have shown promising results in human OSA [(161) (LOE 1a, OEG A)] and the expression of PD-1 ligand in canine OSA correlates with metastasis and T-cell infiltration [(162) (LOE 4b, OEG C)]. Gilvetmab, a caninized PD-1 inhibitor, received conditional licensure in December 2019 in the US for the treatment of canine melanoma and mast cell tumors. Initial trials with a PDL-1 inhibitor in dogs have indicated a positive immune response in OSA [(163) (LOE 2b, OEG B)], although to date improvements in overall survival or disease free interval have not been documented; the development of OX40 agonists is another innovative approach [(164) (LOE 5, OEG D)].

Cell-based therapies and immunomodulation techniques are emerging in OSA treatment. One approach comprising the combination of autologous cancer cell vaccination, adoptive T-cell transfer, and interleukin-2 administration has induced long-term survival in some dogs with OSA [(165) (LOE 2b, OEG B)]. This ELIAS Animal Health treatment received full approval from the US Department of Agriculture Center for Veterinary Biologics (USDA-CVB) in late 2023. Inhaled recombinant human IL-15 for pulmonary metastases from OSA represents a novel methodology [(166) (LOE 2b, OEG B)]. The use of conditionally replicative adenoviral vectors has also been described [(167) (LOE 2b, OEG B)].

Recent work has created an atlas of circulating leukocytes in healthy and OSA-affected dogs, offering valuable insights for future therapies [(168) (LOE 4c, OEG C)]. The safety and survival benefit of oncolytic vesicular stomatitis virus in dogs with naturally occurring OSA [(169) (LOE 2b, OEG B)] opens doors for viral-based therapies.

These significant advancements in immunotherapy are contributing to a deeper understanding of canine OSA and offer hope for improved management and survival.

### Treatment: recommendations

- The combination of limb amputation and adjuvant chemotherapy has repeatedly been shown to achieve the best outcomes for dogs with appendicular limb OSA. Carboplatin as a sole agent is the adjuvant chemotherapy of choice.
- In cases of pathological fracture or extreme pain, amputation alone is an excellent palliative care option.
- Limb sparing is appropriate for a minority of cases. Owners must be apprised of the very high risk of complication, including the risk of limb-construct failure necessitating amputation or euthanasia.

### Treatment: opinions

- Chemotherapy should be initiated shortly after or at the time of amputation to achieve better outcomes (acknowledging that histology will not yet be reported).
- Attitudes toward limb amputation vary among oncologists, general practitioners and pet owners. Several resources are available to help owners better understand the expected functional outcome of amputation and appreciate its potential benefits—these include videos featuring 3 legged dogs, owner testimonials and communication platforms to contact owners of previously treated dogs.
- Osteoarthritis is rarely a contraindication for limb amputation. Neurological deficits indicative of cervical intervertebral disc disease are a contraindication for forelimb amputation.
- The role of anti-NGF monoclonal antibody therapy is under-investigated in canine OSA.
- Although multiple studies demonstrate absence of a measurable survival benefit in dogs receiving aminobisphosphonates as palliative treatment, anecdotal evidence suggests a clinical benefit in a small proportion of cases. Unfortunately, there is currently no predictive marker for this.

## Consideration of prognostic indicators

Dogs with OSA treated with standard of care (local tumor control and adjuvant chemotherapy) have an approximately 40% likelihood of survival at 1 year and 20% at 2 years [(120) (LOE 2b, OEG B)]. About 12% of dogs have gross metastasis at diagnosis [(170) (LOE 2c, OEG B)]. Within cases without metastasis at diagnosis, there is a subset of cases that may develop metastasis early in the course of the treatment and have shorter survival. High serum ALP and body weight were found to be associated with an increased hazard of metastasis development. Increased serum ALP was associated with shorter DFI and ST [(59–61, 63, 81, 171) (LOE 2b–4b, OEG B)] while dogs of lower body weight (<40 kg) had significantly longer DFI and ST [(63, 118, 156) (LOE 2b–4b, OEG B)]. Proximal humerus, scapula, distal femur, and proximal tibia locations, and older age at diagnosis, have been linked with increased mortality [(64, 172) (LOE 2c–4b, OEG B)]. Meta-analysis has specifically identified elevated ALP and proximal humerus location as consistent negative prognostic indicators [(66) (LOE 3a, OEG B)].

When dogs surviving more than a year were studied, only infection of the surgical site after limb-sparing surgery conferred a significantly improved prognosis [(173) (LOE 4a, OEG C)]. Interestingly, surgical site infection after amputation did not influence survival [(174) (LOE 4a, OEG C)].

A summary of clinical and histopathological predictors or prognosticators of outcome for dogs with OSA can be found in Table 3.

**Table 3:** Prognostic factors for canine OSA.

Prognostic factor	Summary/interpretation(s)
Lung metastasis	Very poor prognosis; grave prognosis if metastasis in lung and another site [(139) (LOE 4a, OEG C)].
Lymph node metastasis	Rare in dogs, but those with lymph node metastasis have significantly poorer prognosis in terms of DFI and ST [(81) (LOE 4a, OEG C)].
Tumor mitotic index	Increased mitotic index reduces DFI [(61, 63) (LOE 2b–4a, OEG B)].
Post-operative infection	Can increase ST after limb-sparing surgery [(187) (LOE 4b, OEG C)].
Tumor size	Increasing tumor size significantly associated with pulmonary metastasis [(8) (LOE 4a, OEG C)] and with time to metastasis [(188) (LOE 2b, OEG B)].
Extension of tumor into adjacent soft tissue	Poor prognosis [(8) (LOE 4a, OEG C)].
Percentage of tumor that is necrotic following neoadjuvant chemotherapy	Significant direct correlation with ST; percentage of tumor that is necrotic predictive for survival [(186) (LOE 4b, OEG C)] and strongly predictive for local tumor control but no correlation with time for metastasis [(189) (LOE 2b, OEG B)].
Histological subtype	Fibroblastic subtype has a more favorable prognosis [(8), (190, 191) (LOE 4a, OEG C)].
Age	Dogs <5 years old have shorter DFI compared with older dogs. The mitotic index is higher in tumors from young dogs [(15, 63, 191) (LOE 2b–4a, OEG B)].
Tumor location: humerus	Dogs with tumors involving the humerus have shorter DFI and ST [(118) (LOE 2b, OEG B)].
Histological grade	Higher grades associated with decreased ST and DFI; grade I and II tumors have a significantly better prognosis relative to grade III [(61) (LOE 4a, OEG C)].
Serum alkaline phosphatase	Increased plasma levels of this enzyme associated with shorter DFI and ST [(59–61, 63, 81, 171) (LOE 2b–4b, OEG B)].
Body weight	Dogs of lower body weight (<40 kg) had significantly longer DFI and ST [(63, 118, 187) (LOE 2b–4b, OEG B)].
Vascular invasion	Reduced DFI [(61) (LOE 4a, OEG C)].

DFI, disease-free interval; ST, survival time.

## Referral considerations

Dogs with suspected OSA may be referred for advanced diagnostics, staging, or treatment. Diagnosis can be achieved with cytology in most cases when needed (referral may be needed for ultrasound-guided sampling and/or CT imaging). Even after being diagnosed, some owners may prefer to be referred to a specialist to discuss prognosis and treatment options.

For staging, traditional diagnostic imaging includes 3-view chest radiographs and long bone radiographs. Whole-body CT scans may not identify bone metastases, but they may be more sensitive for detection of small pulmonary nodules. Single positron-emission CT (SPECT) is more efficacious in detecting metastatic lesions and may have an impact on the prognosis, but accessibility to this technology is presently extremely limited [(74, 77) (LOE 4a–5, OEG C)].

### Referral considerations: recommendation

- Advanced surgical techniques such as limb-sparing surgery or clinical trials are reasons to recommend referral.

### Referral considerations: opinions

- If chemotherapy would normally be performed at a referral center, given the evidence supporting early implementation of chemotherapy following amputation, referral for surgery and chemotherapy should be considered to avoid unwanted delay.
- Referral is likely to provide owners with a clearer understanding of their pet’s disease process and options for management and should therefore be considered in all cases where achieving the best outcome overrides other non-medical considerations.
